# Author Correction: Cross-population GWAS and proteomics improve risk prediction and reveal mechanisms in atrial fibrillation

**DOI:** 10.1038/s41467-025-64765-5

**Published:** 2025-10-03

**Authors:** Shuai Yuan, Jie Chen, Xixin Ruan, Yuying Li, Sarah A. Abramowitz, Lijuan Wang, Fangyuan Jiang, Ying Xiong, Michael G. Levin, Benjamin F. Voight, Dipender Gill, Stephen Burgess, Agneta Åkesson, Karl Michaëlsson, Xue Li, Scott M. Damrauer, Susanna C. Larsson

**Affiliations:** 1 Department of Surgery, University of Pennsylvania Perelman School of Medicine, Philadelphia, PA USA; 2 Unit of Cardiovascular and Nutritional Epide-miology, Institute of Environmental Medicine, Karolinska Institutet, Stockholm, Sweden; 3 Department of Medical Epidemiology and Biostatistics, Karolinska Institutet, Stockholm, Sweden; 4 Corporal Michael J. Crescenz VA Medical Center, Philadelphia, PA USA; 5 Department of Gastroenterology, Central South University Third Xiangya Hospital, Changsha, Hunan China; 6 Xiangya School of Public Health, Central South University, Changsha, Hunan China; 7 School of Public Health, Zhejiang University School of Medicine, Hangzhou, Zhejiang China; 8 Division of Cardiovascular Medicine, Perelman School of Medicine, University of Pennsylvania, Philadelphia, PA USA; 9 Department of Genetics, University of Pennsylvania, Philadelphia, PA USA; 10 Department of Systems Pharmacology and Translational Therapeutics, University of Pennsylvania, Philadelphia, PA USA; 11 Institute for Translational Medicine and Therapeutics, University of Pennsylvania, Philadelphia, PA USA; 12 Department of Epidemiology and Biostatistics, School of Public Health, Imperial College London, London, UK; 13 MRC Biostatistics Unit, University of Cambridge, Cambridge, UK; 14 Department of Public Health and Primary Care, University of Cambridge, Cambridge, UK; 15 Medical Epidemiology, Department of Surgical Sciences, Uppsala University, Uppsala, Sweden

Correction to: *Nature Communications* 10.1038/s41467-025-61720-2, published online 11 July 2025

In the version of the article initially published, there was a typo in the Methods “Cross-population GWAS meta-analysis” section, where in the text now reading “Eight studies … contributed to the discovery analysis for the European GWAS, comprising 192,851 atrial fibrillation (AF) cases and 1,239,541 controls,” 192,851 was originally written as “117,905.” In the Fig. 1 text now reading “Cross-ancestry GWAS meta-analysis of 252,438 cases,” 252,438 replaces the original “168,007” cases. The changes have been made in the HTML and PDF versions of the article.

