# Supplementary material for: Cross-population GWAS and proteomics improve risk prediction and reveal mechanisms in atrial fibrillation
Source: Nat Commun. 2025 Jul 11;16:6426. doi: 10.1038/s41467-025-61720-2 (PMC12254421; doi:10.1038/s41467-025-61720-2)
Supplement: Supplementary file 1 — Supplementary Information [file 41467_2025_61720_MOESM1_ESM.pdf]

Supporting materials for

Cross-population GWAS and proteomics improve risk prediction and reveal mechanisms in atrial fibrillation

Shuai Yuan, Jie Chen, Xixin Ruan, Yuying Li, Sarah Abramowitz, Lijuan Wang, Fangyuan Jiang, Ying Xiong, Michael G Levin, Benjamin F. Voight, Dipender Gill, Stephen Burgess, Agneta Åkesson, Karl Michaëlsson, Xue Li, Scott M. Damrauer, Susanna C. Larsson

Table of Contents

Supplementary Methods 3

1. Study participants, phenotyping, genotyping, and quality control 3

2. Mendelian randomization analysis 7

3. Proteins included in the protein score derived from LASSO method 7

Figure S1. The associations between minor allele frequency (MAF) and log-transformed odds ratio (BETA) of atrial fibrillation (AF) for 493 genetic loci among Europeans 8

Figure S2. Scatter plot depicting the effect of genetic instruments for body mass index ratio on atrial fibrillation risk 9

Figure S3. Scatter plot depicting the effect of genetic instruments for waist-to-hip ratio on atrial fibrillation risk 10

Figure S4. Scatter plot depicting the effect of genetic instruments for visceral adiposity on atrial fibrillation risk 11

Figure S5. Scatter plot depicting the effect of genetic instruments for childhood body mass index on atrial fibrillation risk 12

Figure S6. Scatter plot depicting the effect of genetic instruments for apolipoprotein A-I on atrial fibrillation risk 13

Figure S7. Scatter plot depicting the effect of genetic instruments for apolipoprotein B on atrial fibrillation risk 14

Figure S8. Scatter plot depicting the effect of genetic instruments for low-density lipoprotein cholesterol on atrial fibrillation risk 15

Figure S9. Scatter plot depicting the effect of genetic instruments for type 2 diabetes on atrial fibrillation risk 16

Figure S10. Scatter plot depicting the effect of genetic instruments for systolic blood pressure on atrial fibrillation risk 17

Figure S11. Scatter plot depicting the effect of genetic instruments for diastolic blood pressure on atrial fibrillation risk 18
Figure S12. Scatter plot depicting the effect of genetic instruments for thyroid-stimulating hormone on atrial fibrillation risk 19
Figure S13. Scatter plot depicting the effect of genetic instruments for smoking initiation on atrial fibrillation risk 20
Figure S14. Scatter plot depicting the effect of genetic instruments for lifetime smoking index on atrial fibrillation risk 21
Figure S15. Scatter plot depicting the effect of genetic instruments for alcohol consumption on atrial fibrillation risk 22
Figure S16. Scatter plot depicting the effect of genetic instruments for leisure screen time on atrial fibrillation risk 23
Figure S17. Scatter plot depicting the effect of genetic instruments for insomnia on atrial fibrillation risk 24
Figure S18. Tissue-specific enrichment using FUMA. 25
Figure S19. Study design of Mendelian randomization analysis of association between circulating proteins and atrial fibrillation risk.. 26
Figure S20. Population breakdown of PMBB participants 27
Supplementary References 28

Supplementary Methods

1. Study participants, phenotyping, genotyping, and quality control

HUNT (The Nord-Trøndelag Health Study): The HUNT survey, which began in 1984, is a population-based health study carried out in Nord-Trøndelag, Norway.[1] To participate in the HUNT Study, informed consent is required, and the study has been approved by the Data Inspectorate and the Regional Ethics Committee for Medical Research in Norway. The genotyping was performed at the Norwegian University of Science and Technology (NTNU) using the Illumina HumanCore Exome v1.0 and v1.1, and quality control was conducted at both the marker and sample levels. Furthermore, variants from the HUNT low-pass genomes were imputed into The Haplotype Reference Consortium (HRC) samples using Minimac3, and only variants with imputation r^2 values exceeding 0.3 were selected for analysis. The genetic associations with atrial fibrillation were estimated by a generalized mixed model including covariates birth year, sex, genotype batch, and principal components 1-4 as implemented in SAIGE.

deCODE: The study collected data on atrial fibrillation at Landspitali, The National University Hospital, in Reykjavik, and Akureyri Hospital (the two largest hospitals in Iceland) from 1987 to 2015. Controls were 358,161 Icelanders recruited through different genetic research projects at deCODE genetics, excluding those in the atrial fibrillation cohort. The study was approved by the Icelandic Data Protection Authority and the National Bioethics Committee of Iceland (no. VSNb2015030021). The research is centered on 15,220 Icelanders who took part in various disease projects at deCODE genetics, and whole-genome sequence data was used for the analysis. The sequencing process employed Illumina standard TruSeq methodology, and the mean depth was 35× (s.d. 8).[2] The identification of autosomal SNPs and indels was done using Genome Analysis Toolkit version 3.4.0, with variants that failed quality control being removed following Genome Analysis Toolkit best practices. The SNPs and indels identified from sequencing were then imputed into 151,677 Icelanders and their close relatives who had been genotyped using Illumina SNP chips (familial imputation). Variants for the meta-analysis were selected based on matching with either the 1000 Genomes Project reference panel (Phase 3) or the Haplotype Consortium reference panel, taking into account factors such as allele, frequency, and correlation matching. To test for the association between SNPs and atrial fibrillation, logistic regression was used. Additionally, LD score regression was employed to adjust for inflation in test statistics due to cryptic relatedness and stratification.

DiscovEHR: The ongoing MyCode Community Health Initiative of the Geisinger Health System, USA contributed to the DiscovEHR collaboration cohort, which is a hospital-based cohort consisting of 58,124 genotyped individuals of European.[3] The Geisinger Institutional Review Board approved the study. Genotyping was conducted on the Human OmniExpress Exome Beadchip at Illumina after aliquots of DNA were sent. Principal component analysis was used to identify individuals of European, and imputation to the HRC reference panel was performed using the Michigan Imputation Server. Variants with imputation r^2 >0.3 and MAF >0.001 were

retained for analysis. The BGEN dosage files were analyzed using BOLT-LMM, and variants were tested for their association with atrial fibrillation under an additive genetic model.

MGI (The Michigan Genomics Initiative): The MGI cohort is a hospital-based study conducted at Michigan Medicine in the United States, which was approved by the Institutional Review Board of the University of Michigan Medical School. Genotyping was carried out at the University of Michigan using the Illumina Human Core Exome v1.0 and v1.1, followed by quality control at the sample and marker level. Variants from the Haplotype Reference Consortium (HRC) reference panel were imputed using the Michigan Imputation Server (refer to URLs), and variants with imputation r^2 >0.3 were retained for further analysis. The association between the variants and atrial fibrillation was assessed using the Firth bias-corrected logistic likelihood ratio test.

AFGen consortium (The Atrial Fibrillation Genetics consortium): The AFGen consortium is composed of 33 studies, mainly consisting of participants with European ancestry.[4] The identification of atrial fibrillation cases involved documented atrial fibrillation on an electrocardiogram or one in-patient or two out-patient diagnoses of atrial fibrillation, while referents were free of atrial fibrillation. Ethics committees or institutional review boards approved the study, and informed consent was obtained from all cases and referents. Each study conducted genotyping and imputation using the 1000 Genomes Project Phase 1 reference panel, with pre- and post-GWAS filtering steps performed for quality control. The meta-analysis of GWAS results was conducted using an inverse-variance-weighted fixed-effects model and the METAL software.

The FinnGen study (R12): The FinnGen research project is a collaboration between public and private entities that integrates genotype data from Finnish biobanks with digital health record data from Finnish health registries. Genotyping was performed using Illumina (Illumina Inc., San Diego, CA, USA) and Affymetrix arrays (Thermo Fisher Scientific, Santa Clara, CA, USA), with genotype calls made using GenCall and zCall algorithms for Illumina and AxiomGT1 algorithm for Affymetrix data. Sample-wise quality control excluded individuals with ambiguous gender, high genotype missingness (>5%), excess heterozygosity (+-4SD), and non-Finnish ancestry. Variant-wise quality control excluded variants with high missingness (>2%), low Hardy-Weinberg equilibrium P-value (<1e-6), and low minor allele count (MAC<3). Imputation of genotypes was performed using the population-specific SISu v4.0 reference panel. Detailed information can be found: https://finngen.gitbook.io/documentation/

SIMPLER cohorts: SIMPLER stands for Swedish Infrastructure for Medical Population-Based Life-Course and Environmental Research, which includes two Swedish cohorts that are the Swedish Mammography Cohort (SMC, initiated in 1987) and the Cohort of Swedish Men (COSM, initiated in 1997). The clinical examinations in sub-cohorts of SMC (SMCC) and COSM (COSMC) were conducted in 2003-2009 and 2010-2019, respectively, which lead to a total of almost 13 500 participants in the two sub-cohorts. In the whole SIMPLER, there were approximately 37 000 participants with genetic data sequenced by Infinium Global Screening Array (Illumina). Proteome profile has been analyzed using the panels CVD II, CVD III and metabolism from Olink

Proteomics using Li/Hep blood plasma. The proteome profile covers the clinical participants, approximately 13,500 participants. The AF diagnostic data were extracted from from the Swedish National Patient Register, which covers nearly all hospital-based inpatient and outpatient care. In COSMC, staff at Eurofins Genomics (Ebersberg, Germany) extracted DNA from 4 ml EDTA whole blood with use of QIAamp DNA Blood Midi Kit" (Cat. No. 51185) from Qiagen (Hilden, Germany). Subsequently, samples were genotyped at Eurofins Genomics with the Illumina Infinium Global Screening Array version 3 (GSAv3; Illumina, San Diego, CA, USA). Sample exclusion filters applied were: (1) samples with discordant sex information when comparing reported sex and sex determined by the X-chromosome; (2) non-European ancestry; (3) heterozygosity outliers -/+3*IQR from Q1/Q3; (4) low sample call rate (<98 %); (5) HWE exact *p*-value $<1 \times 10{-}7$ (--hwe midp); (6) minor allele count <20; (7) markers not present in 1000G/HRC with matching alleles; (8) allele frequency difference > 0.15 compared with 1000G/HRC. We imputed data by use of chr1-22,X: Michigan Imputation Server v1.2.4 using Eagle v2.4 + minimac v4 and both 1000G phase3 (v5) in tgp.ph 3/ and HRC v1.1 in in hrc1.1/ as reference panels. The final genetic dataset included approximately 7.8 million markers. In SMCC, staff at the Biobank at Karolinska Institutet extracted DNA from 400 µl EDTA whole blood with the Chemagen STAR DNA Blood 400 kit (Perkin Elmer, Waltham, MA, USA) using a ChemagicStar-robot (Hamilton, Reno, NV, USA) based on magnetic bead separation. Subsequently, samples were genotyped at the SNP&SEQ Technology Platform, Science for Life Laboratory, Uppsala University with the Illumina Infinium Global Screening Array Multiple Disease version 1 (GSAv1; Illumina, San Diego, CA, USA). We used the same exclusion filters for quality control. For imputation of chr1-22 the Michigan Imputation Server v1.0.4 using Eagle v2.3 + minimac v3 was used and for chrX the Michigan Imputation Server v1.2.4 using Eagle v2.4 + minimac v4, with reference panels 1000G phase3 (v5) in tgp.ph 3/ and HRC v1.1 in in hrc1.1/. The final genetic dataset included approximately 7.8 million markers. The genetic associations with atrial fibrillation were estimated by a generalized mixed model as implemented in SAIGE.

MVP (Million Veteran Program): The MVP is one of the largest biobanks in the world, established by the U.S. Department of Veterans Affairs to study the genetic and environmental factors influencing health and disease. The MVP was initiated in 2011 by recruiting participants aged 19 to over 100 years from 63 Veterans Affairs Medical Centers across the United States. The program has enrolled a large number of U.S. veterans, with participants providing blood samples for genetic analysis, detailed electronic health record (EHR) data, and self-reported lifestyle and environmental information. Recruitment occurs through VA medical centers, where veterans volunteer to participate, sign informed consent, and complete baseline surveys. For genome-wide association studies, MVP utilizes genotyping arrays, followed by imputation using reference panels to maximize genomic coverage. Rigorous quality control measures are applied, including filtering for call rate, Hardy-Weinberg equilibrium, relatedness, and population structure. Phenotyping is primarily based on EHR data, leveraging structured diagnosis codes, medication records, and clinical notes, often complemented by natural language processing to refine case definitions. In this study, MVP included 635,969 individuals comprising the four groups defined by genetic similarity to the 1000 Genomes Project AFR,

AMR, EAS, and EUR reference superpopulations. GWAS associations were adjusted for age, sex, and top 10 population-specific genetic principal components.

The UK Biobank study: The UK Biobank is a large-scale biomedical database and research initiative, aiming to support diverse and groundbreaking scientific discoveries to improve human health. The study has recruited approximately 500,000 UK individuals aged 40-69 years between 2006 and 2010 and collected extensive genetic and phenotypic information. The study received approval from the North West-Haydock Research Ethics Committee (REC reference: 21/NW/0157), and all participants provided electronic consent. Initially, participants lacking genetic data were excluded. Given few cases in non-European populations, we restricted our analysis to individuals of White-British descent, necessitating the exclusion of participants from other ethnic backgrounds. Participants were genotyped using two similar genotyping arrays designed specifically for the UK Biobank (Applied Biosystems UK BiLEVE Axiom Array and UK BioBank Axiom Array). Phasing and imputation were performed by the UK Biobank analysis team, utilizing the HRC reference panel and UK10K haplotype resource. We performed the GWAS analysis using regenie. GWAS association tests adjusted for age, sex, the first 10 principal components, and genotyping batch.

Biobank Japan: The Biobank Japan (BBJ) is one of the largest population-based biobanks in East Asia, established to investigate the genetic and environmental factors contributing to common diseases. BBJ has enrolled over 200,000 participants from hospitals across Japan, primarily individuals diagnosed with various diseases, making it a hospital-based biobank rather than a purely population-based cohort. Participants provided blood samples, clinical measurements, and lifestyle information, with their medical records linked to Japan's national health system for long-term follow-up. BBJ utilizes genotyping arrays, followed by imputation using ancestry-matched reference panels to improve genetic coverage. Stringent quality control (QC) steps are applied, including filtering for call rate, population outliers, Hardy-Weinberg equilibrium, and relatedness. Detailed information on participants, genotyping, imputation, GWAS analysis can be found in the original paper.[5] GWAS data are publicly available from https://humandbs.dbcls.jp/en/hum0014-v5

Genes & Health: The Genes & Health study is a large-scale, community-based genetic research initiative focused on British Bangladeshi and British Pakistani populations in the UK, two groups that have historically been underrepresented in genetic studies. The study aims to investigate genetic influences on health and disease, particularly in relation to high rates of type 2 diabetes, cardiovascular disease, and consanguinity-related genetic disorders. Recruitment occurs through community outreach, healthcare partnerships, and direct participant engagement, with over 100,000 volunteers providing saliva or blood samples for genetic analysis, alongside access to linked electronic health records (EHRs) and self-reported health information. DNA is extracted from the Oragene (DNA Genotek) saliva system and stored. Genotyping was performed using the Illumina Infinium Global Screening Array v3.0 (with an additional 46 662 Multi-Disease variants). Detailed information on this cohort can be found in the cohort profile paper.[6] GWA data can be downloaded from https://console.cloud.google.com/storage/browser/genesandhealth_publicdatasets/results_G

WAS_public/2024_02_version008phenotypes_binarytraits_51kGSA_TOPMED-r3/3digitICD10?pageState=(%22StorageObjectListTable%22:(%22f%22:%22%255B%255D%22))

2. Mendelian randomization analysis

Employing genetic variants as instrumental variables for an exposure, Mendelian randomization (MR) analysis is an epidemiological approach that can reinforce causal inference in an exposure-outcome association using observational genetic data.[7] The approach can minimize residual confounding because genetic variants are randomly allocated at conception and thus generally unrelated to confounders, such as environmental and self-adopted factors. The random allocation of effect allele in MR design resembles the randomization process in randomized controlled trials. In addition, the method can diminish reverse causation because genetic variants used to proxy the effect of the exposure cannot be modified by the onset and progression of the outcome. There are 3 important assumptions of MR analysis. The first assumption is that the genetic variants proposed as instrumental variables should be robustly associated with the exposure; the second assumption indicates that the used genetic variants should not be associated with any confounders; and the third assumption is that the selected genetic variants should affect the risk of the outcome merely through the risk factor, not via alternative pathways.

3. Proteins included in the protein score derived from LASSO method

After LASSO regression, 87 proteins were selected, including ADGRG2, ADM, AGRN, ANGPTL1, B4GAT1, BCAN, BCHE, CA12, CALCA, CCL18, CCN3, CD1C, CDCP1, CDH2, CDHR5, CEACAM5, CHI3L1, CKAP4, CLC, CLSTN2, COL9A1, CTHRC1, CTSL, CTSV, CXCL17, DCN, EDA2R, EDN1, ELN, F10, F7, FABP3, FABP6, FCRLB, FGFBP1, FUT3_FUT5, GALNT10, GAS6, GDF15, GFRA1, HAVCR1, HSPB6, IFI30, IGDCC4, IL15, IL1RL2, IL22, KLK4, LRRN1, LRTM2, LTBP2, LY96, MAMDC2, MICB_MICA, MMP12, MYOM3, NCAN, NECTIN4, NEFL, NELL2, NFASC, NPL, NPPB, NTproBNP, PRSS2, PRTN3, REN, RET, S100P, SFRP1, SLAMF1, SLC39A14, SLITRK1, SMPD1, SPINK1, SSC4D, SUSD2, THBS2, TIMP4, TNFRSF11B, TNFRSF8, TNFSF12, TNFSF13B, TNNI3, UPB1, VEGFD, XG.

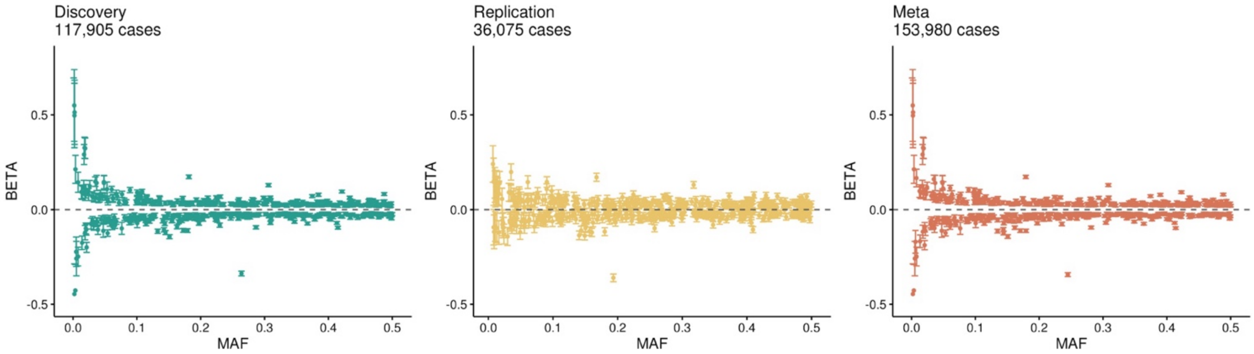

Supplementary Figure 1. The associations between minor allele frequency (MAF) and log-transformed odds ratio (BETA) of atrial fibrillation (AF) for 493 genetic loci among Europeans. The discovery data set included 117,905 AF cases and 1,239,541 controls from eight studies. The replication data set included 36,075 cases and 371,874 controls from the UK Biobank study. The meta-analysis included 153,980 cases and 1,611,415 controls.

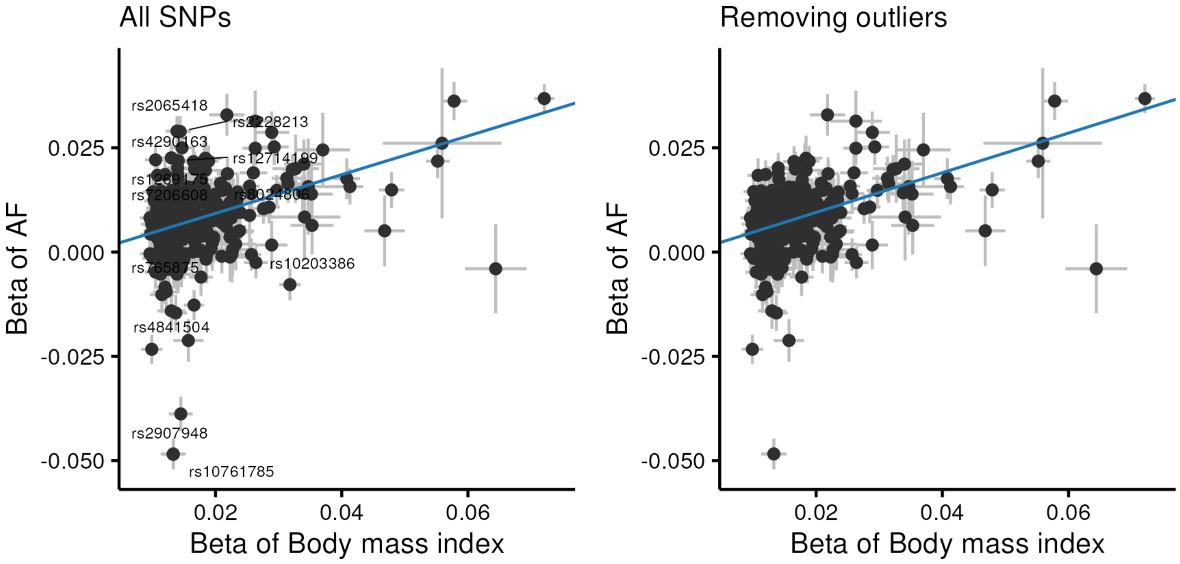

Supplementary Figure 2. Scatter plot depicting the effect of genetic instruments for body mass index ratio on atrial fibrillation risk. SNPs, single nucleotide polymorphisms. Outliers were identified by MR-PRESSO analysis and labeled in the plot. The blue line shows the result of the inverse-variance weighted analysis under a random-effects model.

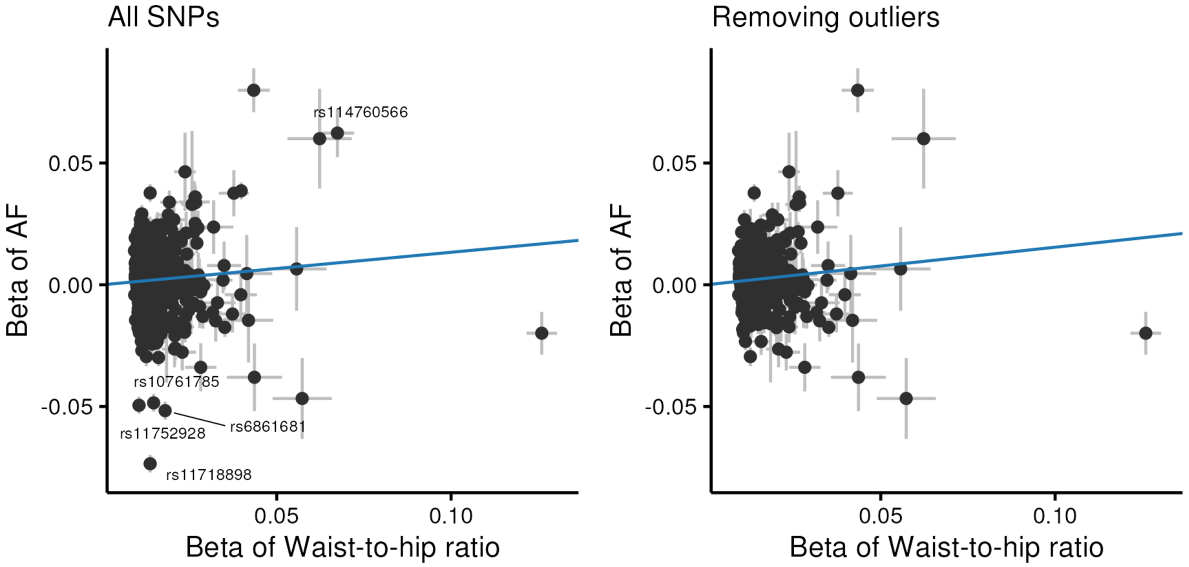

Supplementary Figure 3. Scatter plot depicting the effect of genetic instruments for waist-to-hip ratio on atrial fibrillation risk. SNPs, single nucleotide polymorphisms. Outliers were identified by MR-PRESSO analysis and labeled in the plot. The blue line shows the result of the inverse-variance weighted analysis under a random-effects model.

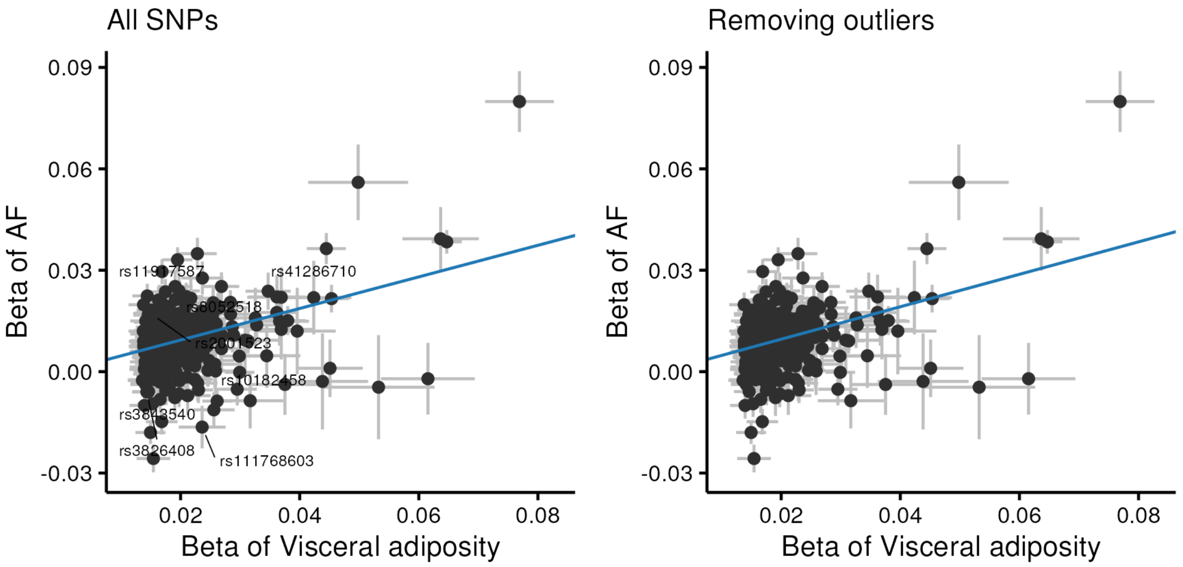

Supplementary Figure 4. Scatter plot depicting the effect of genetic instruments for visceral adiposity on atrial fibrillation risk. SNPs, single nucleotide polymorphisms. Outliers were identified by MR-PRESSO analysis and labeled in the plot. The blue line shows the result of the inverse-variance weighted analysis under a random-effects model.

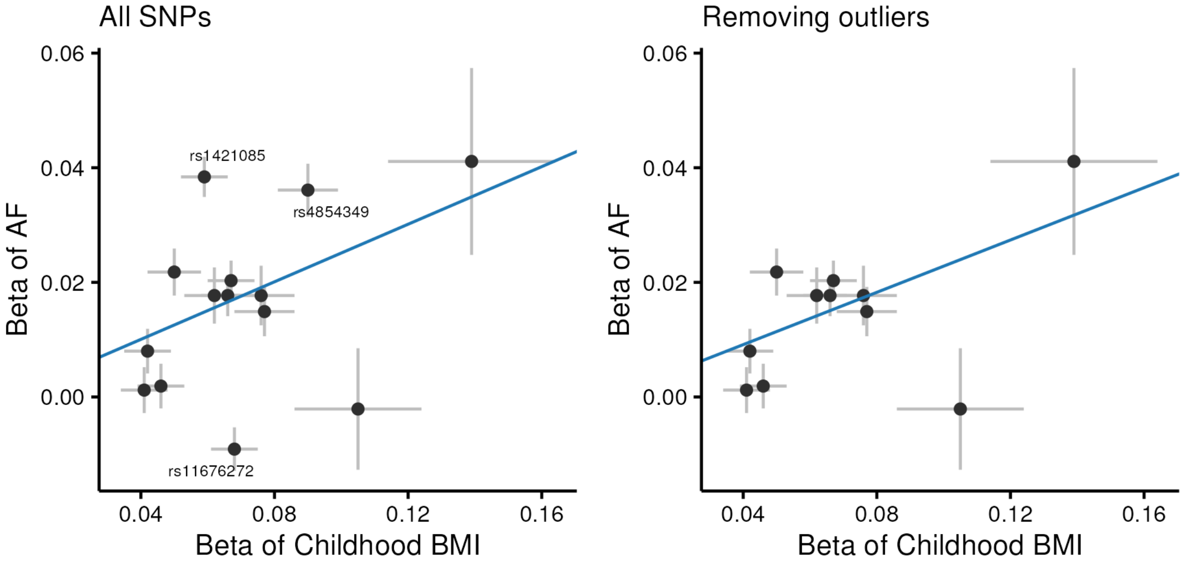

Supplementary Figure 5. Scatter plot depicting the effect of genetic instruments for childhood body mass index on atrial fibrillation risk. SNPs, single nucleotide polymorphisms. Outliers were identified by MR-PRESSO analysis and labeled in the plot. The blue line shows the result of the inverse-variance weighted analysis under a random-effects model.

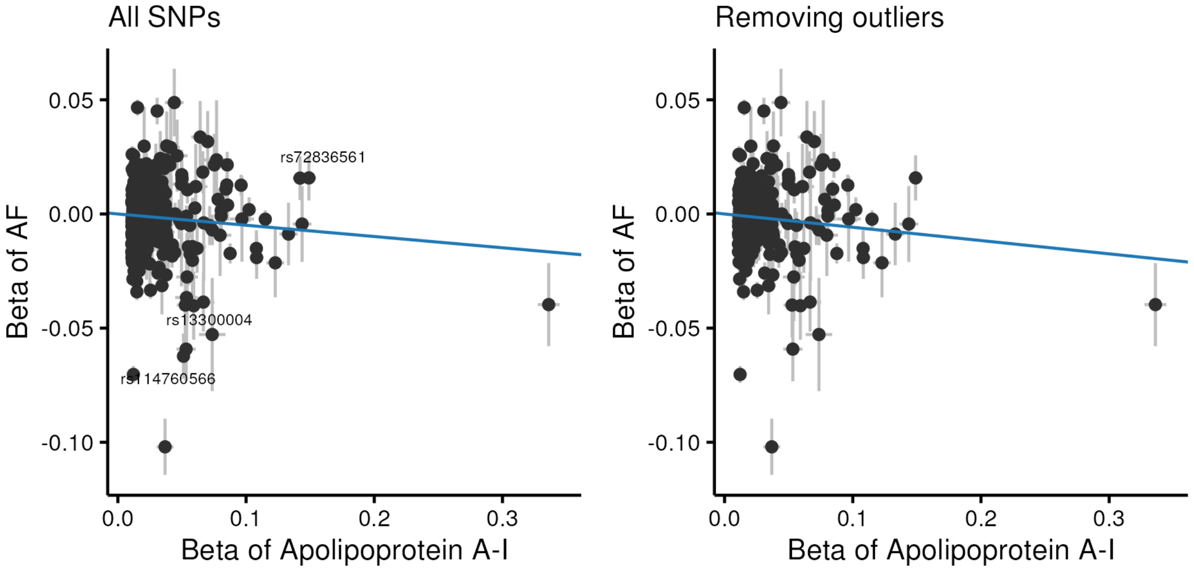

Supplementary Figure 6. Scatter plot depicting the effect of genetic instruments for apolipoprotein A-I on atrial fibrillation risk. SNPs, single nucleotide polymorphisms. Outliers were identified by MR-PRESSO analysis and labeled in the plot. The blue line shows the result of the inverse-variance weighted analysis under a random-effects model.

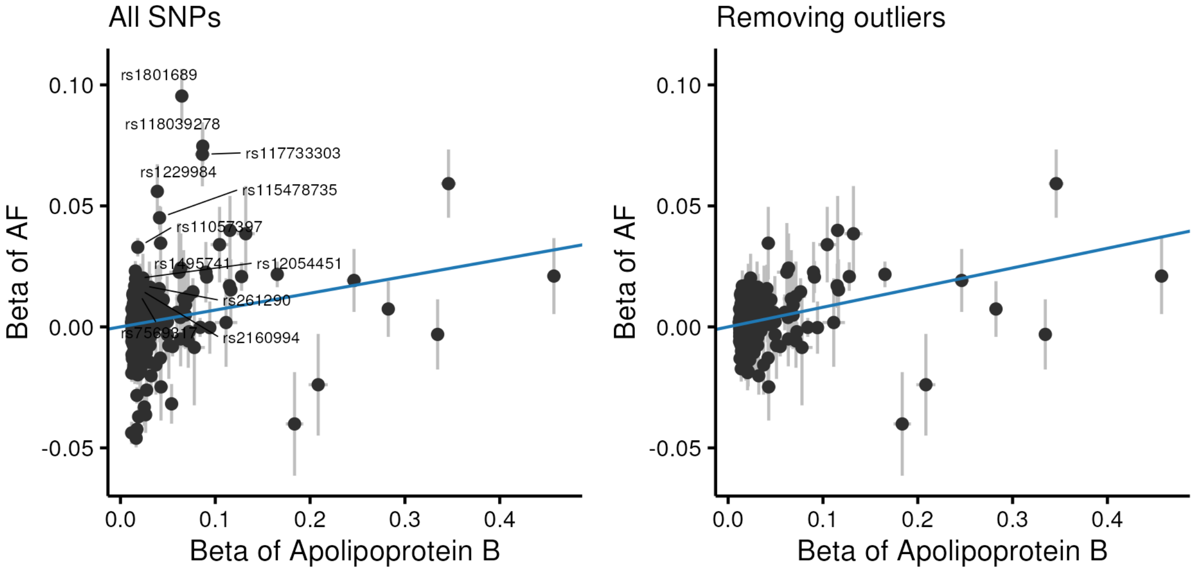

Supplementary Figure 7. Scatter plot depicting the effect of genetic instruments for apolipoprotein B on atrial fibrillation risk. SNPs, single nucleotide polymorphisms. Outliers were identified by MR-PRESSO analysis and labeled in the plot. The blue line shows the result of the inverse-variance weighted analysis under a random-effects model.

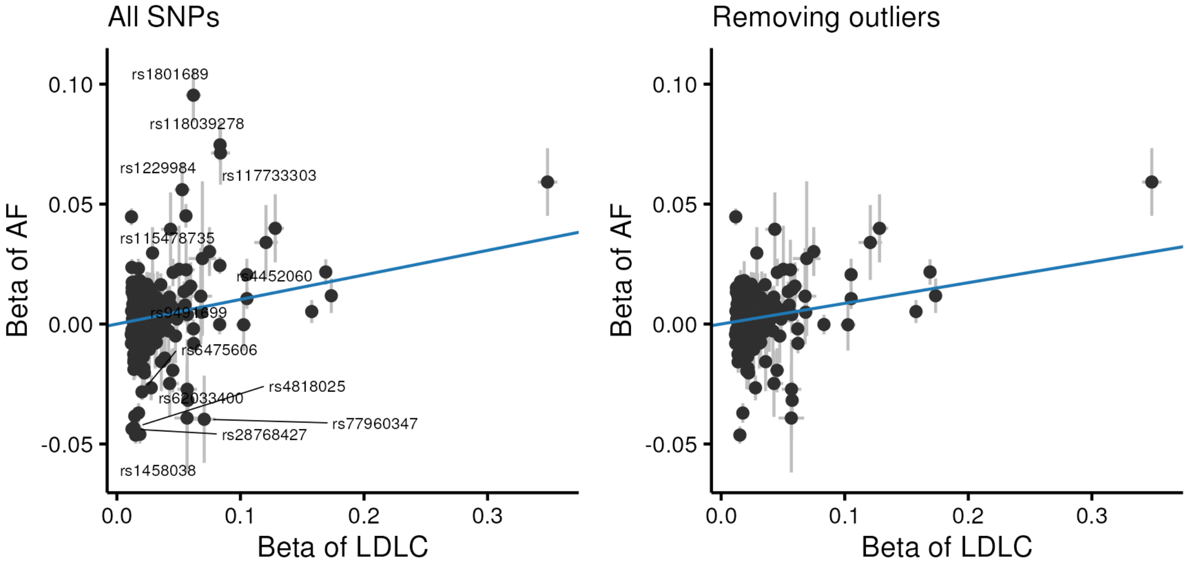

Supplementary Figure 8. Scatter plot depicting the effect of genetic instruments for low-density lipoprotein cholesterol on atrial fibrillation risk. SNPs, single nucleotide polymorphisms. Outliers were identified by MR-PRESSO analysis and labeled in the plot. The blue line shows the result of the inverse-variance weighted analysis under a random-effects model.

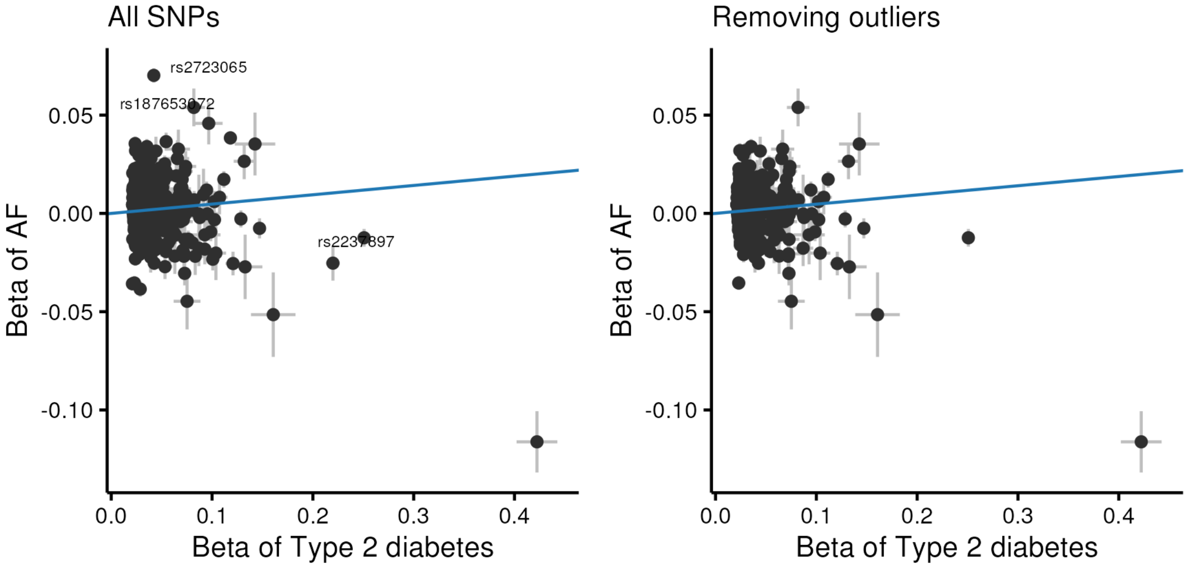

Supplementary Figure 9. Scatter plot depicting the effect of genetic instruments for type 2 diabetes on atrial fibrillation risk. SNPs, single nucleotide polymorphisms. Outliers were identified by MR-PRESSO analysis and labeled in the plot. The blue line shows the result of the inverse-variance weighted analysis under a random-effects model.

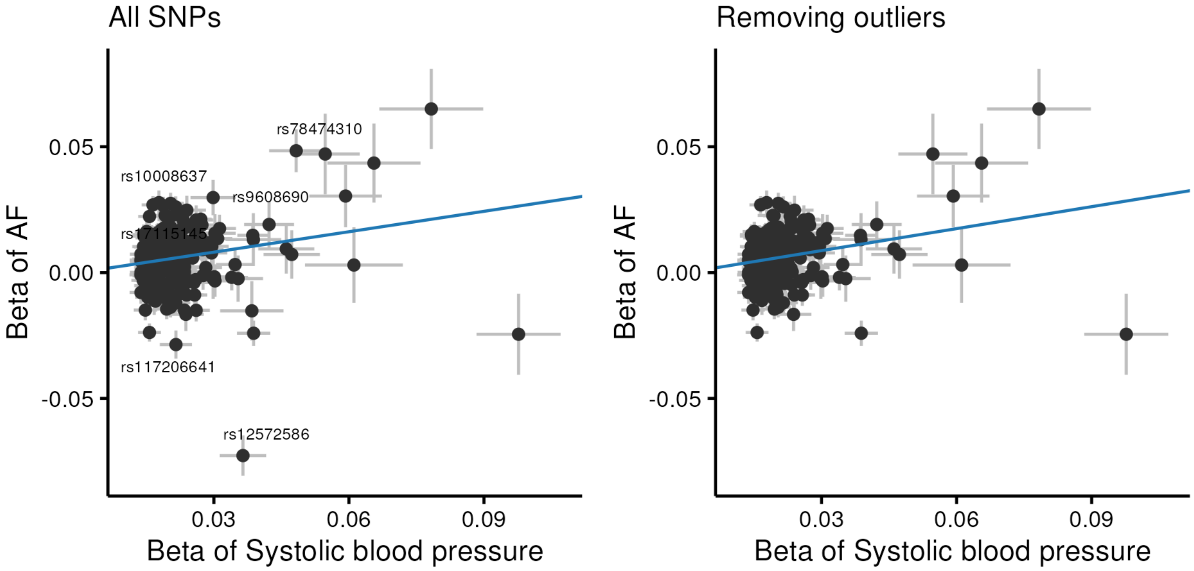

Supplementary Figure 10. Scatter plot depicting the effect of genetic instruments for systolic blood pressure on atrial fibrillation risk. SNPs, single nucleotide polymorphisms. Outliers were identified by MR-PRESSO analysis and labeled in the plot. The blue line shows the result of the inverse-variance weighted analysis under a random-effects model.

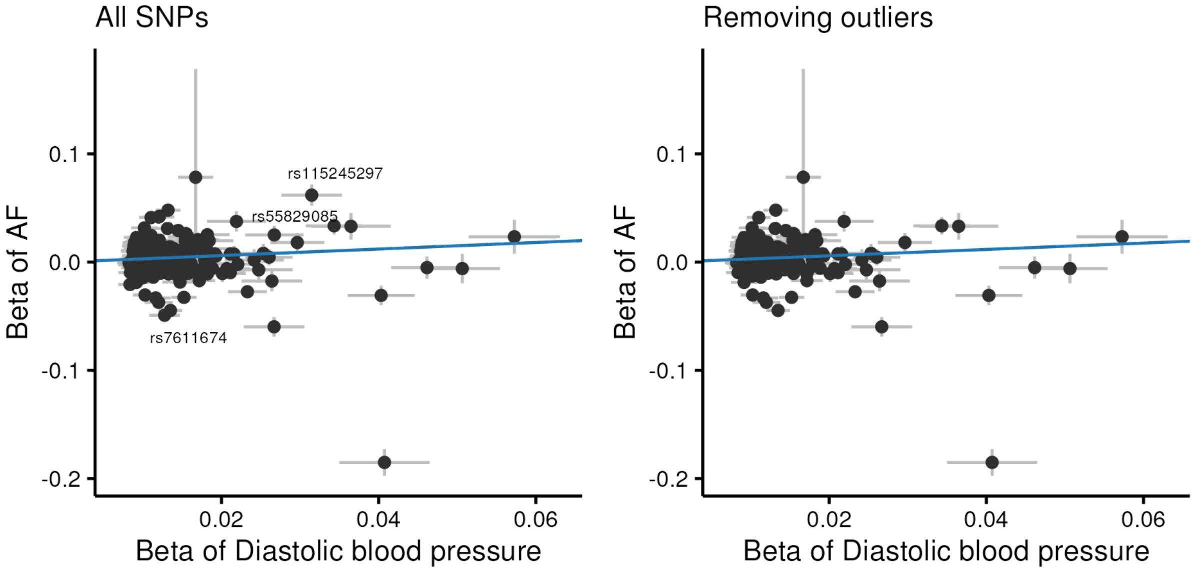

Supplementary Figure 11. Scatter plot depicting the effect of genetic instruments for diastolic blood pressure on atrial fibrillation risk. SNPs, single nucleotide polymorphisms. Outliers were identified by MR-PRESSO analysis and labeled in the plot. The blue line shows the result of the inverse-variance weighted analysis under a random-effects model.

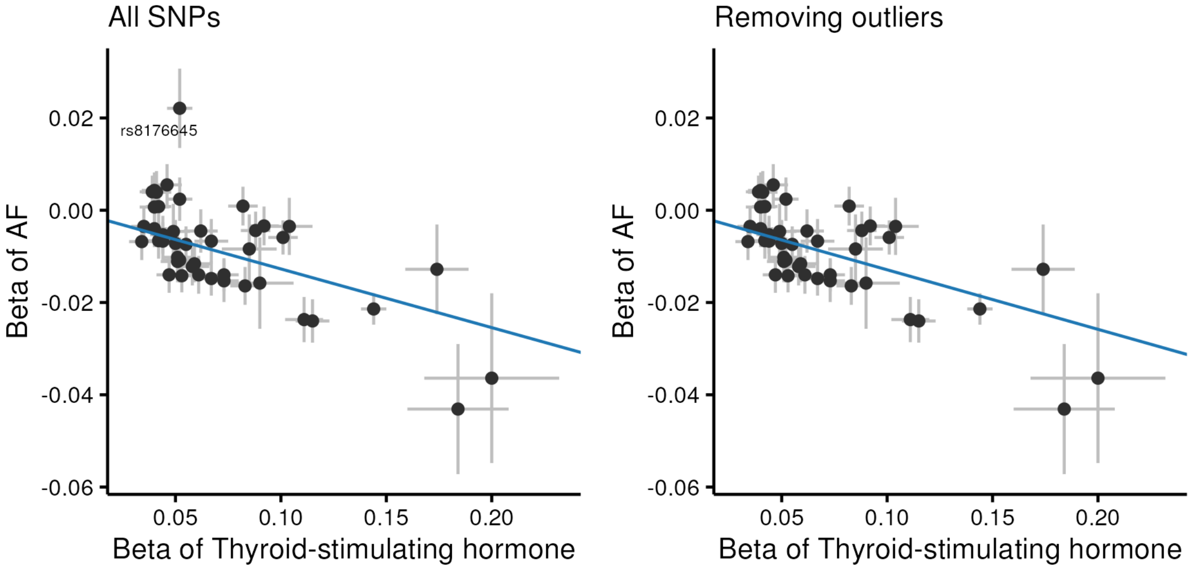

Supplementary Figure 12. Scatter plot depicting the effect of genetic instruments for thyroid-stimulating hormone on atrial fibrillation risk. SNPs, single nucleotide polymorphisms. Outliers were identified by MR-PRESSO analysis and labeled in the plot. The blue line shows the result of the inverse-variance weighted analysis under a random-effects model.

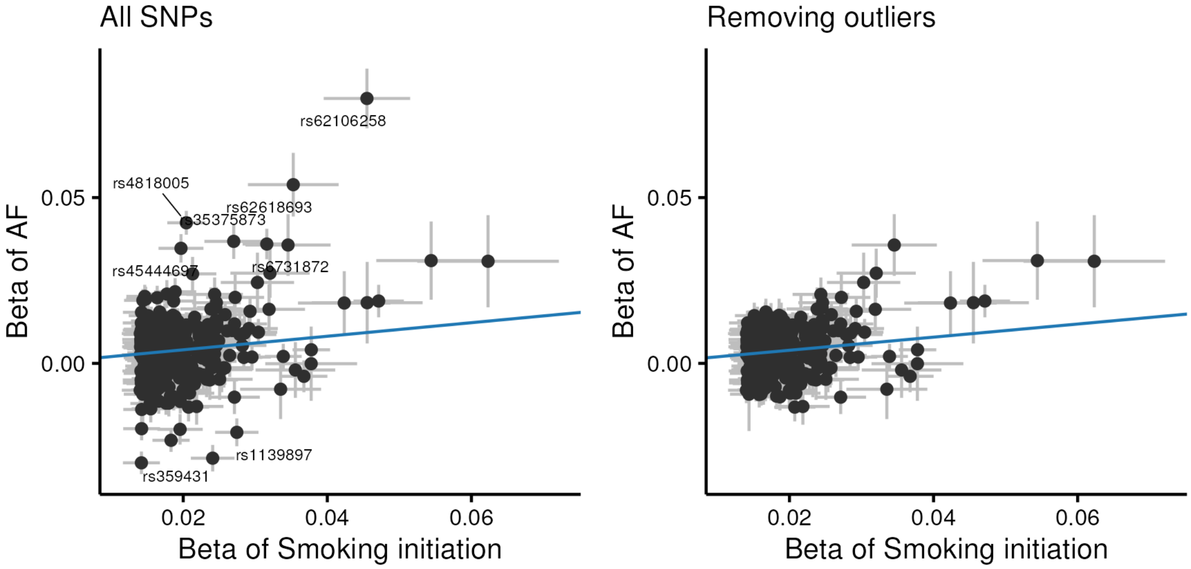

Supplementary Figure 13. Scatter plot depicting the effect of genetic instruments for smoking initiation on atrial fibrillation risk. SNPs, single nucleotide polymorphisms. Outliers were identified by MR-PRESSO analysis and labeled in the plot. The blue line shows the result of the inverse-variance weighted analysis under a random-effects model.

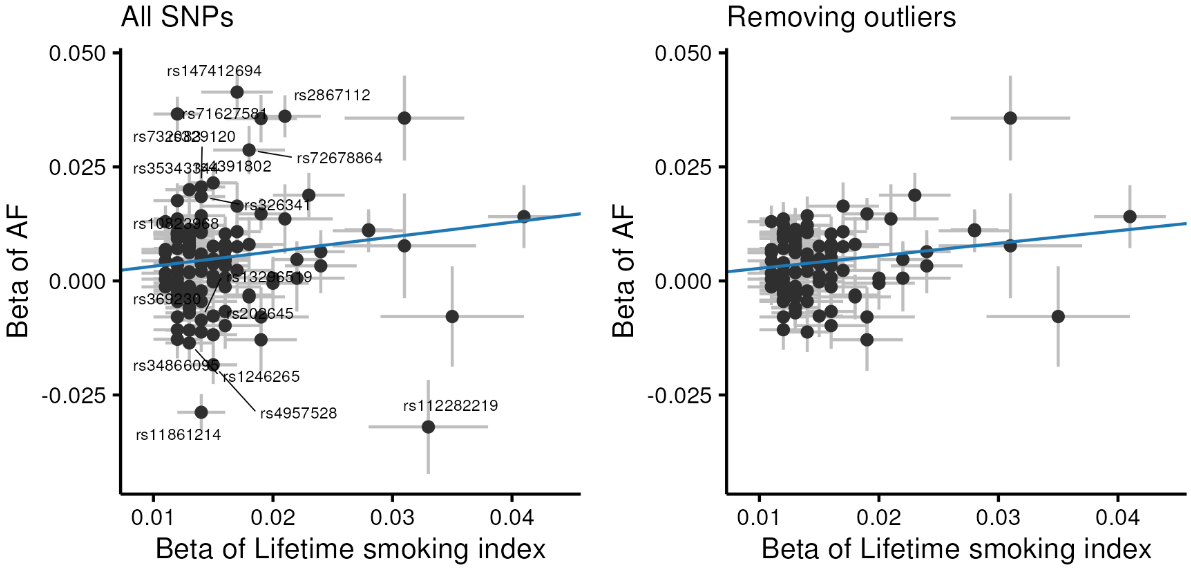

Supplementary Figure 14. Scatter plot depicting the effect of genetic instruments for lifetime smoking index on atrial fibrillation risk. SNPs, single nucleotide polymorphisms. Outliers were identified by MR-PRESSO analysis and labeled in the plot. The blue line shows the result of the inverse-variance weighted analysis under a random-effects model.

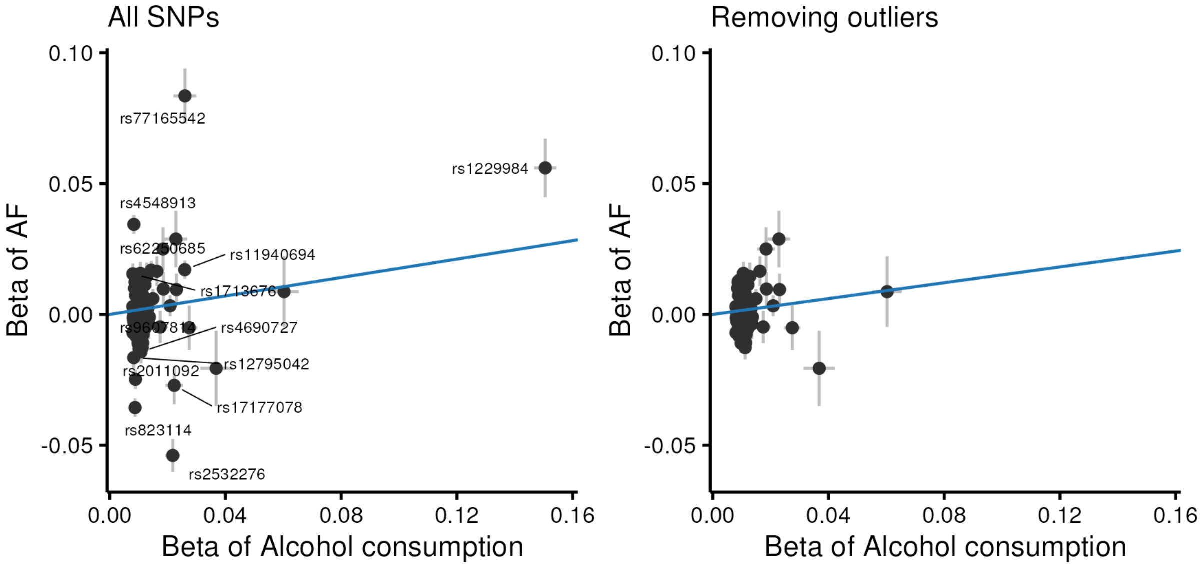

Supplementary Figure 15. Scatter plot depicting the effect of genetic instruments for alcohol consumption on atrial fibrillation risk. SNPs, single nucleotide polymorphisms. Outliers were identified by MR-PRESSO analysis and labeled in the plot. The blue line shows the result of the inverse-variance weighted analysis under a random-effects model.

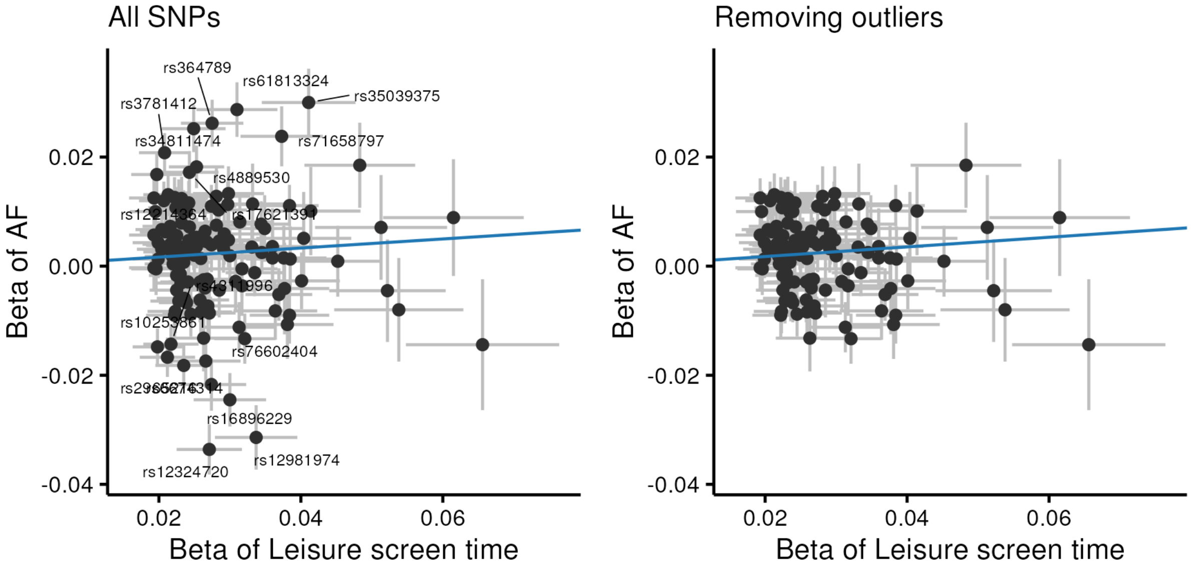

Supplementary Figure 16. Scatter plot depicting the effect of genetic instruments for leisure screen time on atrial fibrillation risk. SNPs, single nucleotide polymorphisms. Outliers were identified by MR-PRESSO analysis and labeled in the plot. The blue line shows the result of the inverse-variance weighted analysis under a random-effects model.

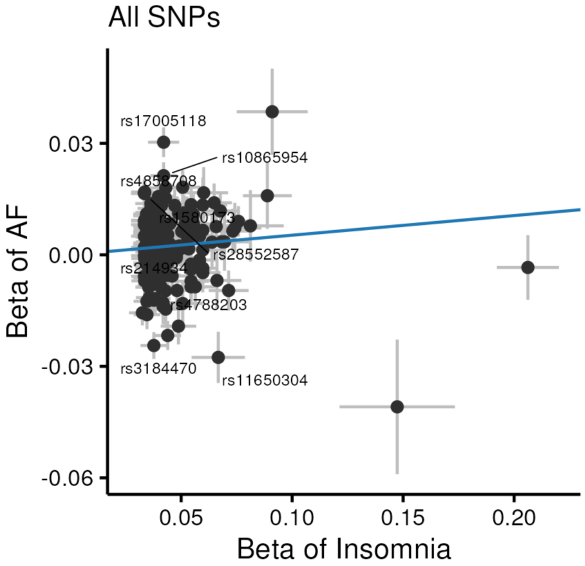

Supplementary Figure 17. Scatter plot depicting the effect of genetic instruments for insomnia on atrial fibrillation risk. SNPs, single nucleotide polymorphisms. Outliers were identified by MR-PRESSO analysis and labeled in the plot. The blue line shows the result of the inverse-variance weighted analysis under a random-effects model.

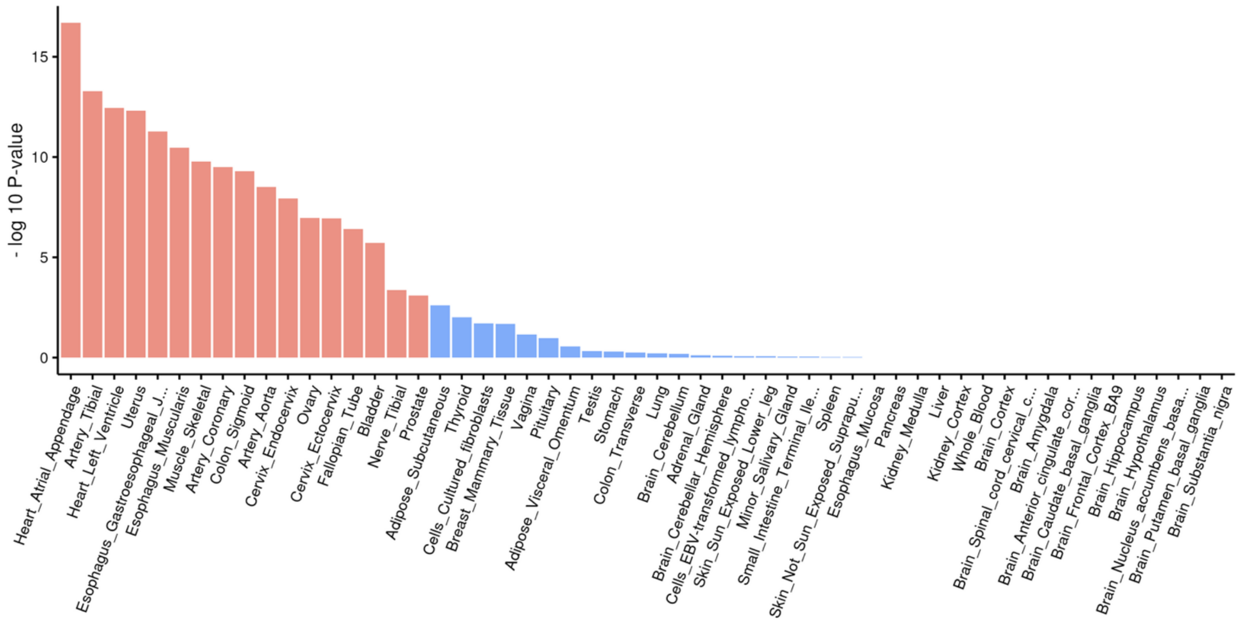

Supplementary Figure 18. Tissue-specific enrichment using FUMA.

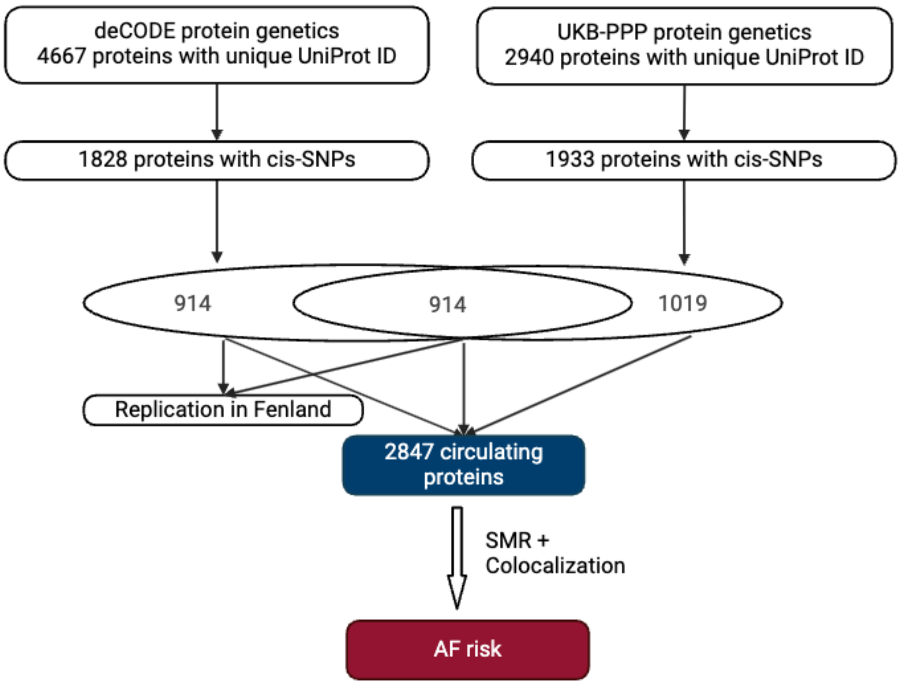

Supplementary Figure 19. Study design of Mendelian randomization analysis of association between circulating proteins and atrial fibrillation risk. SMR, Summary-data-based Mendelian Randomization SNPs, single nucleotide polymorphisms.

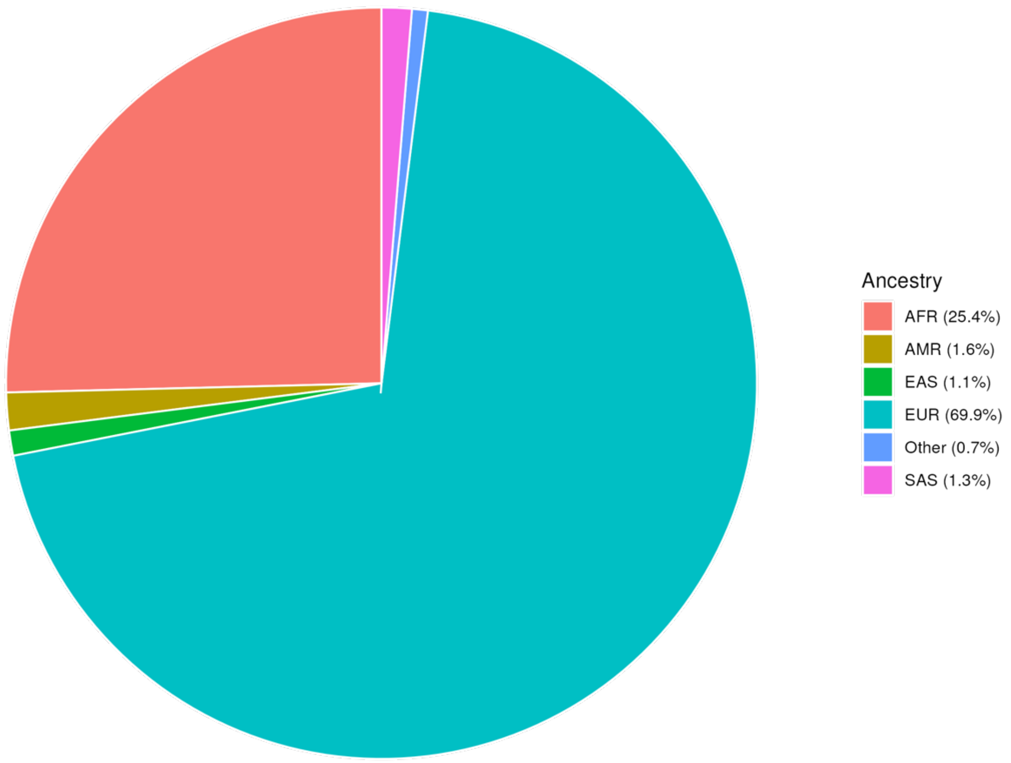

Supplementary Figure 20. Population breakdown of PMBB participants.

Supplementary References

1. Krokstad S, Langhammer A, Hveem K, et al. Cohort Profile: the HUNT Study, Norway. *Int J Epidemiol*. 2013;42(4):968-977. doi:10.1093/ije/dys095

2. Gudbjartsson DF, Helgason H, Gudjonsson SA, et al. Large-scale whole-genome sequencing of the Icelandic population. *Nat Genet*. 2015;47(5):435-444. doi:10.1038/ng.3247

3. Carey DJ, Fetterolf SN, Davis FD, et al. The Geisinger MyCode community health initiative: an electronic health record-linked biobank for precision medicine research. *Genet Med*. 2016;18(9):906-913. doi:10.1038/gim.2015.187

4. Christophersen IE, Rienstra M, Roselli C, et al. Large-scale analyses of common and rare variants identify 12 new loci associated with atrial fibrillation. *Nat Genet*. 2017;49(6):946-952. doi:10.1038/ng.3843

5. Sakaue S, Kanai M, Tanigawa Y, et al. A cross-population atlas of genetic associations for 220 human phenotypes. *Nat Genet*. 2021;53(10):1415-1424. doi:10.1038/s41588-021-00931-x

6. Finer S, Martin HC, Khan A, et al. Cohort Profile: East London Genes & Health (ELGH), a community-based population genomics and health study in British Bangladeshi and British Pakistani people. *Int J Epidemiol*. 2020;49(1):20-21i. doi:10.1093/ije/dyz174

7. Burgess S, Thompson SG. *Mendelian Randomization: Methods for Causal Inference Using Genetic Variants*. Second edition. CRC Press; 2021.
