# Supplementary material for: Cross-population GWAS and proteomics improve risk prediction and reveal mechanisms in atrial fibrillation
Source: Nat Commun. 2025 Jul 11;16:6426. doi: 10.1038/s41467-025-61720-2 (PMC12254421; doi:10.1038/s41467-025-61720-2)
Supplement: Supplementary file 2 — Description of Additional Supplementary Files [file 41467_2025_61720_MOESM2_ESM.pdf]

Description of Additional Supplementary Files

Supplementary Data 1. Data sources

Supplementary Data 2. Genetic loci associated with atrial fibrillation among Europeans

Supplementary Data 3. Genetic loci associated with atrial fibrillation in cross-population GWAS meta-analysis

Supplementary Data 4. Gene prioritization

Supplementary Data 5. Pathway enriched using Reactome data base

Supplementary Data 6. Pathway enriched using GO data base

Supplementary Data 7. Genetic correlation between atrial fibrillation and other circulatory diseases among Europeans in MVP

Supplementary Data 8. Genetic correlation between atrial fibrillation and other circulatory diseases among Africans in MVP

Supplementary Data 9. Results of Mendelian randomization analysis of 37 modifiable risk factor for atrial fibrillation

Supplementary Data 10. Results of Summary-based Mendelian randomization analysis of 2847 circulating proteins for atrial fibrillation

Supplementary Data 11. Results of colocalization for 28 proteins associated with atrial fibrillation

Supplementary Data 12. Results of Summary-based Mendelian randomization analysis of 22 circulating proteins for atrial fibrillation in the Fenland replication

Supplementary Data 13. Results of Summary-based Mendelian randomization analysis of 22 circulating proteins for atrial fibrillation in the Fenland replication

Supplementary Data 14. Genetically predicted atrial fibrillation in relation to protein levels in deCODE

Supplementary Data 15. Genetically predicted atrial fibrillation in relation to protein levels in UKB-PPP

Supplementary Data 16. Incidence and hazard ratios of atrial fibrillation across deciles of protein score

Supplementary Data 17. Incidence and hazard ratios of atrial fibrillation across deciles of polygenic risk score

Supplementary Data 18. Data sources for exposures used in the Mendelian randomization analysis.

Supplementary Data 19. Genetic instruments for modifiable risk factors in relation to atrial fibrillation

Supplementary Data 20. Genetic instruments for circulating proteins and associations with atrial fibrillation

Supplementary Data 21. Associations of plasma proteins with incident atrial fibrillation
