# Supplementary material for: Cross-population GWAS and proteomics improve risk prediction and reveal mechanisms in atrial fibrillation
Source: Nat Commun. 2025 Jul 11;16:6426. doi: 10.1038/s41467-025-61720-2 (PMC12254421; doi:10.1038/s41467-025-61720-2)
Supplement: Supplementary file 24 — Reporting Summary [file 41467_2025_61720_MOESM24_ESM.pdf]

nature portfolio

Corresponding author(s): Shuai Yuan

Last updated by author(s): Jun 27, 2025

Reporting Summary

Nature Portfolio wishes to improve the reproducibility of the work that we publish. This form provides structure for consistency and transparency in reporting. For further information on Nature Portfolio policies, see our Editorial Policies and the Editorial Policy Checklist.

Statistics

For all statistical analyses, confirm that the following items are present in the figure legend, table legend, main text, or Methods section.

n/a	Confirmed	
☐	☒	The exact sample size (n) for each experimental group/condition, given as a discrete number and unit of measurement
☐	☒	A statement on whether measurements were taken from distinct samples or whether the same sample was measured repeatedly
☐	☒	The statistical test(s) used AND whether they are one- or two-sided<br>*Only common tests should be described solely by name; describe more complex techniques in the Methods section.*
☐	☒	A description of all covariates tested
☐	☒	A description of any assumptions or corrections, such as tests of normality and adjustment for multiple comparisons
☐	☒	A full description of the statistical parameters including central tendency (e.g. means) or other basic estimates (e.g. regression coefficient) AND variation (e.g. standard deviation) or associated estimates of uncertainty (e.g. confidence intervals)
☐	☒	For null hypothesis testing, the test statistic (e.g. F, t, r) with confidence intervals, effect sizes, degrees of freedom and P value noted<br>*Give P values as exact values whenever suitable.*
☐	☒	For Bayesian analysis, information on the choice of priors and Markov chain Monte Carlo settings
☒	☐	For hierarchical and complex designs, identification of the appropriate level for tests and full reporting of outcomes
☒	☐	Estimates of effect sizes (e.g. Cohen's d, Pearson's r), indicating how they were calculated

Our web collection on statistics for biologists contains articles on many of the points above.

Software and code

Policy information about availability of computer code

Data collection	No software was used.
Data analysis	SAIGE 0.44.6.4, regenie v4.0, Plink V2, GWASinspector 1.7.3, METAL latest version, LDSC v1.0.1, MAGMA 2.9.0, FUMA, S-MultiXcan, R 4.3.2, SMR

For manuscripts utilizing custom algorithms or software that are central to the research but not yet described in published literature, software must be made available to editors and reviewers. We strongly encourage code deposition in a community repository (e.g. GitHub). See the Nature Portfolio guidelines for submitting code & software for further information.

Data

Policy information about availability of data

All manuscripts must include a data availability statement. This statement should provide the following information, where applicable:

- Accession codes, unique identifiers, or web links for publicly available datasets
- A description of any restrictions on data availability
- For clinical datasets or third party data, please ensure that the statement adheres to our policy

The UK Biobank data are available through application (https://www.ukbiobank.ac.uk/). The summary-level data of FinnGen (https://storage.googleapis.com/finngen-public-data-r12/summary_stats/release/finngen_R12_I9_AF.gz) and MVP (https://ftp.ncbi.nlm.nih.gov/dbgap/studies/phs002453/analyses/GIA/) are publicly online. The GWAS data generated in this study have been deposited in the NHGRI-EBI GWAS catalog database under accession code GCST90624411, GCST90624412, GCST90624413, and GCST90624414. Source data are provided with this paper.

Research involving human participants, their data, or biological material

Policy information about studies with human participants or human data. See also policy information about sex, gender (identity/presentation), and sexual orientation and race, ethnicity and racism.

Reporting on sex and gender	Sex was self-reported as well as genetically determined. The analyses were performed in both men and women. No-sex specific analysis was performed.
Reporting on race, ethnicity, or other socially relevant groupings	The study design was specifically tailored to consider the crucial factors of race, ethnicity, and ancestry. These factors play a significant role in understanding the genetic variations and their implications, making them essential components of our research. Harmonizing Genetic Ancestry and Self-identified Race/Ethnicity (HARE) groups were used to define race/ethnicity. Briefly, HARE enhances classification by integrating self-identified race/ethnicity (SIRE) and genetically inferred ancestry (GIA). HARE ensures accurate classification by using GIA to refine and, if necessary, impute SIRE, improving the reliability of race/ethnicity assignment in genetic research.
Population characteristics	The average age of participants in MVP is 61.7 years. The average age of participants in UK Biobank is 56.5 years. The median age of participants in SIMPLER is 56-59 years. The average age of participants in FinnGen is 53 years. The number of cases and controls is reported in Table S1.
Recruitment	Participants recruitment has been specified in the paper Method part.
Ethics oversight	The study complied with all relevant regulations governing the use of human participants and was conducted in accordance with the principles of the Declaration of Helsinki. Participants in the FinnGen study provided informed consent for biobank research, with the study protocol (No. HUS/990/2017) approved by the Coordinating Ethics Committee of the Hospital District of Helsinki and Uusimaa (HUS). The UK Biobank received ethical approval from the North West Multi-centre Research Ethics Committee (approval number: 11/NW/0382), with all participants giving informed consent. The Million Veteran Program (MVP) was approved by the VA Central Institutional Review Board (IRB), and participants provided informed consent. All included studies adhere to rigorous ethical guidelines to ensure the protection of participants and the integrity of the research.

Note that full information on the approval of the study protocol must also be provided in the manuscript.

Field-specific reporting

Please select the one below that is the best fit for your research. If you are not sure, read the appropriate sections before making your selection.

☒ Life sciences ☐ Behavioural & social sciences ☐ Ecological, evolutionary & environmental sciences

For a reference copy of the document with all sections, see nature.com/documents/nr-reporting-summary-flat.pdf

Life sciences study design

All studies must disclose on these points even when the disclosure is negative.

Sample size	The sample size is predetermined in HUNT, deCODE, MGI, DiscoverEHR, AFGen, FinnGen, MVP, BioBank Japan, Genes & Health. After excluding non-Europeans and low-quality data, the sample size was determined in SIMPLER and UK Biobank. We did not calculate the desired sample size; however, it is one of the largest GWAS until now.
Data exclusions	Participants were removed due to low data quality.
Replication	For European GWAS, UK Biobank was used as an independent replication. Of the 493 loci, 479 displayed consistent effect estimates between the discovery and replication datasets.
Randomization	Not relevant given no randomization was performed in the study.
Blinding	Not relevant given that the current study is not a type of study that requires blinding.

Reporting for specific materials, systems and methods

We require information from authors about some types of materials, experimental systems and methods used in many studies. Here, indicate whether each material, system or method listed is relevant to your study. If you are not sure if a list item applies to your research, read the appropriate section before selecting a response.

Materials & experimental systems

n/a	Involved in the study
☒	☐ Antibodies
☒	☐ Eukaryotic cell lines
☒	☐ Palaeontology and archaeology
☒	☐ Animals and other organisms
☒	☐ Clinical data
☒	☐ Dual use research of concern
☒	☐ Plants

Methods

n/a	Involved in the study
☒	☐ ChIP-seq
☒	☐ Flow cytometry
☒	☐ MRI-based neuroimaging

Plants

Seed stocks	*Report on the source of all seed stocks or other plant material used. If applicable, state the seed stock centre and catalogue number. If plant specimens were collected from the field, describe the collection location, date and sampling procedures.*
Novel plant genotypes	*Describe the methods by which all novel plant genotypes were produced. This includes those generated by transgenic approaches, gene editing, chemical/radiation-based mutagenesis and hybridization. For transgenic lines, describe the transformation method, the number of independent lines analyzed and the generation upon which experiments were performed. For gene-edited lines, describe the editor used, the endogenous sequence targeted for editing, the targeting guide RNA sequence (if applicable) and how the editor was applied.*
Authentication	*Describe any authentication procedures for each seed stock used or novel genotype generated. Describe any experiments used to assess the effect of a mutation and, where applicable, how potential secondary effects (e.g. second site T-DNA insertions, mosiacism, off-target gene editing) were examined.*
